# Social Adaptation in Context: The Differential Role of Religiosity and Self-Esteem in Vulnerable vs. Non-vulnerable Populations – A Registered Report Study

**DOI:** 10.3389/fpsyg.2021.519623

**Published:** 2021-11-24

**Authors:** Alejandra Neely-Prado, Michiel van Elk, Gorka Navarrete, Fernanda Hola, David Huepe

**Affiliations:** ^1^Center for Social and Cognitive Neuroscience, School of Psychology, Universidad Adolfo Ibáñez, Santiago, Chile; ^2^Cognitive Psychology Unit, Department of Psychology, Leiden University, Leiden, Netherlands; ^3^Department of Psychology, Pontificia Universidad Católica de Chile, Santiago, Chile

**Keywords:** religiosity, self-esteem, social adaptation, vulnerable contexts, moderated mediation

## Abstract

There is evidence that religiosity and self-esteem are positively related, while self-esteem and religiosity in turn predict successful social adaptation. Moreover, self-esteem has been shown to be directly related to social adaptation in vulnerable contexts. In this registered report study, we tested the hypothesis that religiosity has a positive influence on social adaptation for people living in vulnerable contexts and that self-esteem is a mediator of this relationship. Evidence from this study indicates that neither there is any effect of religiosity on social adaptation nor on self-esteem, independent of whether people live in vulnerable contexts or not.

## Introduction

The implications of the effects of religiosity on mental health and other psychosocial variables have been widely discussed (Zinnbauer et al., 1997; Hackney and Sanders, 2003; Bonelli and Koenig, 2013). It has been suggested that religiosity may affect well-being and mental health through its influence on positive emotions (Van Cappellen et al., 2017). Self-esteem, i.e., having a positive view and evaluation of oneself and one’s capabilities, could be a potential mediator of this relationship, as it has been found to be positively associated with religiosity in several studies (Smith et al., 1964; Benson and Spika, 1973; Watson et al., 1985; Sherkat and Reed, 1992; Plante and Boccaccini, 1997; Greenway et al., 2003; Krause, 2003). It has been proposed that religiosity offers experiences of divine support, which in turn bolsters self-esteem through a sense of self-worth (Schieman et al., 2017). Religiosity has been shown, for instance, to ameliorate the repercussions of childhood poverty on self-esteem (Henderson, 2016). High self-esteem, in turn, protects from the negative effects of poverty and stress (Henderson, 2016; Moksnes et al., 2016; Neely-Prado et al., 2019). For example, self-esteem has been shown to attenuate the negative effects of poverty on hippocampal gray matter volume (Wang et al., 2016). At the same time, it seems to foster several well-being and mental health outcomes across samples with different characteristics (Dumont and Provost, 1999; Orth and Robins, 2014; von Soest et al., 2018).

Religion could specifically have a facilitatory effect on self-esteem and mental health for vulnerable populations that have only limited resources for coping, maintaining a positive self-image, and for successful social adaptation. Specific mechanisms through which religiosity fosters self-esteem have been proposed, which are as follows: (1) religion could provide people with a specific social identity and meaning, through religious rituals and norms. This generates a positive bias toward the ingroup that strengthens the self-esteem of its members (Tajfel, 1981; Brown, 2000). Since religious meaning gives at least two reference points—the material and non-material dimensions of life—it is proposed to minimize psychological loss when the self is perceived as not meeting certain group expectations, which also protects self-esteem (Carvalho, 2018). Another mechanism that suggests positive effects of religiosity on self-esteem is (2) the Terror Management theory that posits self-esteem as an evolved cultural tool for dealing with the anxiety of facing the knowledge of death and uncertainty (Greenberg et al., 1997). This theory suggests that religiosity can offer a symbolic and/or literal immortality through a sense of the value of the person for a certain group or through beliefs in an afterlife.

Either way, religiosity seems to ameliorate negative evaluations that could occur from real-life interactions and social support (Sherkat and Reed, 1992; Greenway et al., 2003; Joshanloo and Daemi, 2014; Carvalho, 2018).

Since vulnerable populations are characterized by complex and unsafe social interactions and little social support, we believe it is a special case to test if the abovementioned relationships will hold. A vulnerable population is here defined as people who typically live under chronic stress, with low income, low access to quality health services, and low education (Ministerio de Desarrollo Social, 2015). In contrast, a non-vulnerable population refers to people with higher socioeconomic status and with access to quality education and health services (Asociación de Investigadores de Mercado, 2018).

Although it can be argued that because religion fosters the most needed sense of protection and security, religiosity levels should be higher in people who live in vulnerable contexts, and we believe that religiosity will be equally present in vulnerable and non-vulnerable populations in Chile. It has been proposed that countries with income inequality tend to have higher levels of religiosity (Rees, 2009); however, it is still unclear if there are also differences between people from those countries who live in rich vs. poor and vulnerable environments. Although no national statistics were found when comparing religiosity according to any socioeconomic variables, we do know that by 2017 only 6% of the Chilean adult population stated itself as a non-believer, 14% said they sometimes believe in God and sometimes not, and 77% said they had no doubts about the existence of God (GFK Adimark, 2017). Another national survey conducted in 2018 showed that 80% of the population stated that they believe in God and always had, 6% of the population believes in God and not always had, 9% of the population does not believe in God although they used to, and 3% of the population has never believed in God (CEP, 2018). In addition, the “high society” of Chile tends to be very conservative, with religious ideas leading to their conservatism (León, 2013). According to this information, it seems reasonable to expect that religiosity will be present across vulnerable and non-vulnerable populations in similar proportions.

In contrast, variation of self-esteem and social adaptation levels is expected in both groups. In this sense, vulnerability does not determine if people will have higher or lower self-esteem and social adaptation; moreover, these are desirable characteristics when living in harsh environments. However, what determines the presence of high self-esteem and high social adaptation? We have seen that high self-esteem serves as social adaptation positively in vulnerable contexts (Neely-Prado et al., 2019). However, it is possible that this will also be the case for people that live in non-vulnerable contexts. High levels of self-esteem foster well-being and related features across samples and groups with different characteristics (Dumont and Provost, 1999; Orth and Robins, 2014; von Soest et al., 2018). Thus, we propose that a differential mechanism between both the groups is the influence of religion on self-esteem, because religiosity is expected to have little variation across groups, we expect that its impact on self-esteem will be different depending on the context people live in (vulnerable vs. non-vulnerable).

We hypothesize that religiosity will have greater indirect effects on social adaptation through self-esteem in the vulnerable group than in a non-vulnerable group because other demographics (e.g., income and education) and personality predictors of self-esteem (Wang and Veugelers, 2008; Heaven and Ciarrochi, 2015) are mostly absent in vulnerable contexts (Duncan and Magnuson, 2013; Kakinami et al., 2015). Living in vulnerable contexts, thus, encompasses several developmental and contextual difficulties (Evans and Kim, 2010; Yoshikawa et al., 2012; Lipina and Segretin, 2015), which makes it harder for people to have an optimal set of psychological tools to adapt and cope. Religiosity may serve as a coping mechanism in people living under important amounts of stress, such as in the case of vulnerable populations and bolstering levels of self-esteem. At the same time, higher levels of self-esteem are expected to serve as a psychological tool so that people living in vulnerable contexts enlarge their chances of achieving social adaptation.

Social adaptation has been defined as the capacity to confront, relate, compromise, and cooperate with the environment and others, accommodating thoughts and behaviors in this process (Samadi and Sohrabi, 2016). Social adaptation involves emotion regulation, search for rewarding social interactions, and higher social sensitivity (Ma et al., 2016); other relevant aspects of social adaptation include coping skills, interpersonal relationships, and skills regarding play and leisure to measure social adaptation among a group of children (Racz et al., 2017). There has been a special interest in how social adaptation varies specifically in vulnerable contexts (Krishnadas et al., 2013; Holz et al., 2015; Tottenham and Galván, 2016), and we would like to contribute to this topic by studying the underlying effects of religiosity and self-esteem on a direct measure of social adaptation in these environments.

People living in vulnerable contexts apparently differ in how they achieve social adaptation, and it is still not fully understood which variables determine these differences. We believe that religiosity could be an important factor through its positive effects on self-esteem. However, most studies about religiosity focus on educated people from developed countries (Fehr and Heintzelman, 1977; Thomson and Jaque, 2014; Vonk and Pitzen, 2017), and currently, we lack a good understanding of the relationship between religion and social adaptation in more vulnerable populations. In Chile particularly, 20.7% of the population lives under conditions of multidimensional poverty (Ministerio de Desarrollo Social, 2018), a category that takes into account access of people to other aspects beyond income, such as education, health, and job conditions (Ministerio de Desarrollo Social, 2015). This population varies in its levels of social adaptation as well (Neely-Prado et al., 2019). Thus, testing the effects of religiosity on social adaptation in a sample of Chilean people who live in vulnerable contexts compared to a non-vulnerable sample could further insight into the psychological mechanisms underlying the effects of religiosity on social adaptation of people when living in contexts characterized by chronic stress and poverty.

Thus, the aim of the present registered report study was to test for the ameliorative effects of religiosity and self-esteem on social adaptation for people living in a harsh environment. We hypothesized that (1) high religiosity will predict high social adaptation through high levels of self-esteem in a vulnerable group and (2) this indirect relationship between religiosity and social adaptation will be reduced or even absent in a non-vulnerable group.

## Methods

### Power Analysis

Although effect sizes vary greatly across studies, overall the reported correlations between religiosity and self-esteem reported by the literature are small (Gebauer et al., 2012; Papazisis et al., 2014). On the other hand, small and medium significant correlations have been reported between self-esteem and socioeconomic status (Twenge and Campbell, 2002; Chung et al., 2014; von Soest et al., 2018). Focusing on small and positive effect sizes (*r* = 0.15, *p* < 0.05), to obtain 0.8 power, a sample of 273 subjects would be required. However, this study includes a subset of data collected by an ongoing broader project that focuses on neither religiosity nor self-esteem but on the association between metacognition and living in vulnerable contexts. For this reason, in regard to the vulnerable sample, we are limited to an already existing sample size (*N* = 243). Data from a non-vulnerable group will be collected specially for this project, and we expect to collect the same number of subjects as in the experimental group, matching participants by age and sex.

### Participants and Procedure

This project is a part of the broader research funded by the Chilean National Commission for Science and Technology (CONICYT/FONDECYT, no. 1201486), where a group of people living in vulnerable contexts was assessed to study different aspects of social adaptation. The present project focused only on data collection from a control group (i.e., non-vulnerable) to test its main hypothesis that encompasses comparing both groups.

For the vulnerable group data collection, several tests and questionnaires were collected in a vulnerable population in neighborhoods characterized by their low socio-economic status (SES). Participants were reached by accessibility and received economic compensation for their participation. Trained social science professionals administered the protocol to each participant at the seat of the neighborhood’s board. The authors only reviewed the descriptives of data collected from the vulnerable group. However, descriptives of the religiosity variable were also reviewed for data collected from the control group. No other revision or data analysis was made specifically with the variables of interest of this study.

The vulnerable sample consists of 243 adults living in contexts characterized by social vulnerability and where conditions of chronic stress are present. This refers to people living in areas of social risk, low socioeconomic levels, and low education levels. The sample was from Santiago, Chile, and was obtained by accessibility.

Criteria for eligibility were being between 18 and 45 years old, not having auditory or visual impairments, and not having a history of any neurological or psychiatric disorder. These criteria were assessed in a selection interview prior to the administration of any test or questionnaire. The Ethics Committee of Universidad Adolfo Ibáñez authorized the complete procedure.

To be sure that each person came from a vulnerable context, we asked their social index card numbers, which give information about their social contexts. We selected only those beneath the 40th percentile of the Chilean welfare program (according to the “social card of households” from the Ministerio de Desarrollo Social, 2018). The Chilean welfare program grants subsidies, bonuses, and other facilities to people who are living in a vulnerable situation. For people or families to receive these benefits, they must be registered to a social index card that indicates the percentile of the vulnerability of the person or family. To calculate these percentiles, their socioeconomic status and the access they have to public services are taken into account. People or families up to the 40th percentile are considered as living in a vulnerable situation, and adults from this group were included in this study.

Since the vulnerability index used for selecting people who live in vulnerable contexts is established by an interview that takes into account several variables and which procedure is not detailed enough for the public, we proposed the following “non-vulnerability” index to serve as inclusion criteria for control data collection: that the income of the person, educational level, and job reach the ABC1 or C2 socio-economic classification (“high SES”) according to the socioeconomic group survey (Asociación de Investigadores de Mercado, 2018). This survey was included for self-report in the online survey.

At pre-registration, it was stated that people from the control group would also be asked for their social index card numbers from the social protection program, and if they had one, its number would be provided as exclusion criteria (only people from vulnerable contexts have the social index card). If the person did not have a social index card, then the classification from the socioeconomic group survey would provide information for socioeconomic inclusion criteria. As there is no access to the complete list of people who have the social card of the social protection program, we would have to rely on self-report. Both elements together, the social index card and the socioeconomic group survey, would assure that our control group corresponds to the 85th percentile or has access to education, quality health services, and sufficient income. However, during the study, we learned that not everybody knows if they are enrolled in the social protection program, which makes it an unreliable source for determining that a person is not from vulnerable contexts. Thus, we decided to use only the classification from the socioeconomic group survey, since we already confirmed from the vulnerable sample that the survey does correctly identify people who live in vulnerable contexts.

Data collection for the control sample was stopped after completing a sample of 243 individuals who qualify as non-vulnerable and match the vulnerable group by age and sex. This sample was obtained by accessibility.

Statistical analyses were conducted only after the collection of the control group data, which was collected through an online survey, such as three of the questionnaires that were also administered to assess the vulnerable population sample and demographic and socioeconomic questions.

The online survey was divulged through social media and messaging apps. Data were collected between February 16, 2021 and June 17, 2021. Except for 132 participants that completed the consent form (first task) but did not follow through, 1,079 answered two or more of the eight tasks. A total of 953 of these participants (88.3%) completed the protocol. After randomly matching by age and sex of the participants to the experimental group, 243 with complete questionnaires remained.

At first, as we had no funding for the data collection of the non-vulnerable group, we decided that we would not compensate participants. Since the present study only requires people answering four surveys online, with no need to meet an interviewer on a schedule or move from home (as they did in the data collection process for the vulnerable sample), and the effort required to answer the online survey is considerably lower. However, we were able to raffle a $200.000 Chilean pesos gift card to help speed up the data collection for the non-vulnerable sample.

People with self-reported visual or auditory impairments and neurological or psychiatric disorders were excluded from the study. This exclusion criterion was employed both in the vulnerable and non-vulnerable groups because people with psychiatric and psychological disorders usually experience social adaptation difficulties, which is normally a diagnosis criterion. This exclusion criterion may have an impact on the results because it could be that low social adaptation is determined by the number of people with psychiatric and psychological disorders in each group. Nonetheless, the main interest of this study lies in psychologically healthy people who depend on their context can struggle more or less with social adaptation.

For the control group data collection, people answered Rosenberg’s Self-Esteem Scale, Santa Clara’s Religiosity Questionnaire, the Social Adaptation Self-Evaluation Scale (SASS), and demographic questions (such as the socioeconomic group survey) used to match the non-vulnerable with the vulnerable group by sex and age. In addition, it allowed us to evaluate inclusion criteria. The online survey was configured so that all fields were mandatory, avoiding missing data. Although at pre-registration, it was stated that no other questionnaires would be added. After the outbreak of COVID, we considered that it could be important to add some questions to control for stress and changes of context during the pandemic, so questions to grasp these variables were added. The complete survey can be accessed with the rest of the documents of this study in https://github.com/gorkang/jsPsychHelpeR-Neely.

By the time data collection for the control sample started, Chile was on its way out from the most recent peak of COVID cases, and free and voluntary vaccines were already being administered from the state. According to the statistics provided by the official health authorities of the country (Ministerio de Salud (MINSAL), 2021c), 16% of the population had already received the first dose. By the end of the data collection process, 74% had received the first dose and 25% had also the second dose. Regarding confirmed cases by February 16, there were 19,644 accumulated deaths related to COVID-19, 2,547 new cases, and a positivity rate of 7.45% (Ministerio de Salud (MINSAL), 2021a). By the end of data collection, there were 31,184 accumulated deaths related to COVID-19, 6,683 new cases, and a positivity rate of 7.82% (Ministerio de Salud (MINSAL), 2021b).

In contrast to the non-vulnerable group data collection, the vulnerable group data collection included 28 evaluations, such as self-report measurements and neuropsychological tests, namely, Matrices of WAIS-IV and INECO Frontal Screening, which measure fluid intelligence and executive functions, respectively.

We do not believe that the other tests and questionnaires included for assessing the vulnerable group would prime their responses and alter their results, because they measured a wide variety of cognitive and psychological variables that are not notably related to questionnaires of interest for the present study. In addition, tests and questionnaires were randomized to avoid this type of issue. The new online survey will include only one measure for each variable of interest (demographic characterization, social adaptation, religiosity, and self-esteem), and presentation order will also be randomized.

For accessibility and economic reasons, data from the non-vulnerable sample were collected through an online survey, although data from the vulnerable group were obtained in a lab context. The main confound that could arise given the differences in data collection methods between the groups could be the attentional aspect of people answering an online survey vs. having a professional accompanying the participants. In addition, any bias that could have been introduced by the social interaction between the professional and the participant will not be present in the second survey (such as positive or negative feelings toward the interviewer and therefore more or less willing to answer the survey attentively and sincerely). However, these biases are present and uncontrolled in most surveys, regardless of the presence of an interviewer. Thus, we believe that it is reasonable to mix both methods.

### Measures

All participants from the vulnerable and non-vulnerable groups completed several questionnaires, among which was a list of demographic questions (age, sex, income, and educational level), the Rosenberg Self-Esteem Scale, the Santa Clara’s Religiosity Scale, and the SASS. All questionnaires were originally published in English, but Spanish versions were needed since Chile is a Spanish-speaking country. The Rosenberg Self-Esteem Scale and SASS have already been validated in Spanish (Bobes et al., 1999; Rojas-Barahona et al., 2009). Santa Clara’s Religiosity Scale had no Spanish validation and was translated by our team (see specifications below).

The SASS (Bosc et al., 1997) assesses motivation and behavior of people implicated in engaging in social activities through 20 items asking about hobbies, family life, work, relationships, intellectual interests, environment managing capacity, and perception of self-performance. Answers range from 0 to 3, where higher scores indicate better social adjustment. Total scores between 35 and 52 points of a total of 60 are considered a normal social adaptation marker. This test has shown high levels of reliability (α = 0.81) and validity (Ueda et al., 2011).

Rosenberg’s Self-Esteem Scale (Rojas-Barahona et al., 2009) was administered to assess the feelings of people toward themselves, measuring the positive or negative valence that results from the evaluation of personal characteristics. It is a 10-item self-report scale; Likert answers ranging from 1 = “strongly disagree” to 4 = “strongly agree” are offered, with a maximum score of 40 points (Swami et al., 2016). This scale has been shown to have good validity and reliability (α = 0.75) in a Chilean sample (Rojas-Barahona et al., 2009).

The Santa Clara Religiosity Scale (Plante and Boccaccini, 1997) was used to assess the strength of religious faith. This measure provides an index of the importance that God has in the lives of people regardless of the specific religious denomination. The scale consists of a 10-item self-report scale, where answers are structured in a Likert format ranging from 1 = “strongly disagree” to 4 = “strongly agree.” This test has been shown to consist of a single dimension and has been shown to be internally consistent (α = 0.93) (Lewis et al., 2001). It has also been shown to be a valid and reliable measure (Plante et al., 1999). To the best of our knowledge, there is no Spanish version of this questionnaire. However, the Centre of Social and Cognitive Neuroscience (CSCN) has created a translated version through a back-translation method.

### Statistical Analysis

Once Stage 1 revision was approved, we created the subset of data from the vulnerable sample collected during the broader project, such as only the self-esteem, social adaptation, religiosity questionnaires, and demographic variables. Then, we eliminated every row that contains missing data in any of the three questionnaires and the sex or age variables. Once we had the clean data, we created a script to match each new participant from the non-vulnerable population to each participant from the vulnerable population by age and sex. Once all participants from the vulnerable population had their match, data collection was stopped.

For the non-vulnerable group data collection, only participants with no missing data were included. Once data collection was completed, total scores were calculated, and statistical analysis was run.

Reliability was evaluated for each scale, using Cronbach’s alpha analysis (Cronbach, 1951). Each one showed good levels of reliability according to the standardized alpha of Cronbach’s coefficient. The SASS had an alpha value of 0.77, the Santa Clara’s Religiosity Scale had an alpha value of 0.96, and the Rosenberg Self-Esteem Scale had an alpha value of 0.85. The most relevant conclusion from these outcomes is that questionnaires performed well but also that the Santa Clara’s Religiosity Scale continued with high levels of reliability after translation into Spanish.

Moreover, descriptive measures were calculated for each scale, such as their means, SDs, variance, range, asymmetry, and kurtosis. To describe the raw relationship between variables, a matrix of Pearson’s correlations was calculated. Statistical analysis was conducted for both samples using R in RStudio (RStudio Team, 2018).

**FIGURE 1:**
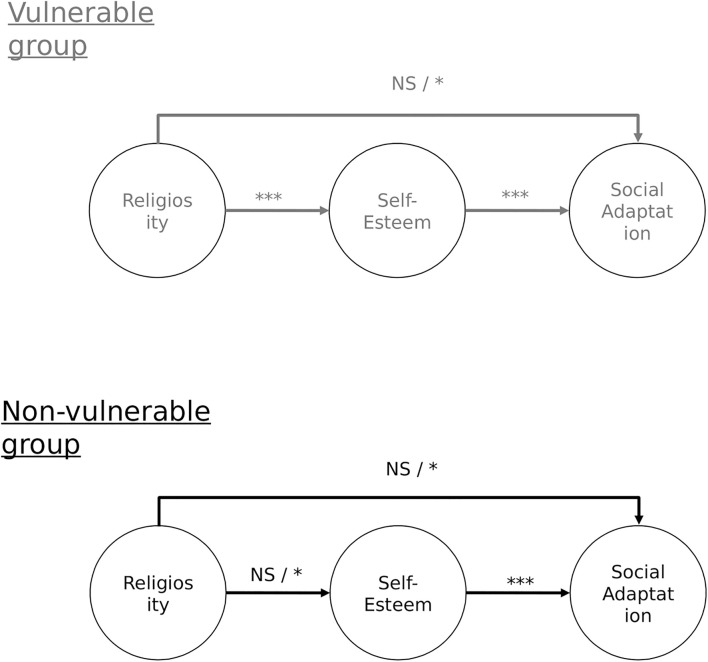
Data for the model in gray were collected before pre-registration. *p < 0.05; **p < 0.01; ***p < 0.001, NS, non-significant.

Finally, to confirm our hypothesis that self-esteem will mediate the relationship between religiosity and social adaptation, but only in people who live in vulnerable contexts, we conducted a moderated mediation analysis (Figure 1). Moderated mediation occurs when the effects of mediation are conditional on the presence of another independent variable (Preacher et al., 2007).

At pre-registration, it was stated that we would use path regression analysis to test moderated mediation following Petty’s model (Petty et al., 1993; Muller et al., 2005; Chen et al., 2016). Demos and Washburn (n.d) recommendations (Washburn, 2018; Demos, 2019) would be followed to conduct a moderated mediation analysis through a multi-group strategy under a structural equation modeling background, by using the “Lavaan” package (Rosseel, 2012; Rosseel et al., 2017). The independent variable would be mean centered, and deviations from normality would be managed through robust estimators, such as weighted least square mean and variance (WLSMV) (Finney and DiStefano, 2006). Given that mediation effects are usually asymmetrical and underpowered a percentile bootstrapping with 10,000 draws would be used to provide reliable estimates (Preacher et al., 2007). The fit of the model would be evaluated following Hu and Bentler’s recommendations of a Root-Mean-Square Error of Approximation (RMSEA) < 0.06, SRMR < 0.08, and CFI and TLI > 0.95 (Cheung and Rensvold, 2002; Chen, 2007; Steiger et al., 2014; Cangur and Ercan, 2015). If the model fits the data well, regression estimates would be interpreted to observe if mediated moderation is present, and whether it is a complete or partial effect. If there is an indirect effect of the independent variable (religiosity) over the dependent variable (social adaptation), through self-esteem holds for the vulnerable group, but not for the non-vulnerable group, there is no evidence to discard our hypothesized model.

## Results

Table 1 shows descriptive statistics by group. Total 56% of participants were women, and 44% were men, and the mean age of the entire sample was 32 years old.

**TABLE 1:** Descriptives for study variables per group.

**Group**	**Variable**	***M***	**SD**	**Min**	**Max**	**Skew**	**Kurtosis**
Experimental	1. Religiosity	22.08	9.01	10	40	0.05	−1.15
	2. Social adaptation	40.52	6.17	18	53	−0.46	0.21
	3. Self-esteem	31.96	5.69	12	40	−0.84	0.52
Control	1. Religiosity	20.64	8.50	10	40	0.46	−0.67
	2. Social adaptation	43.98	6.16	18	58	−0.53	0.76
	3. Self-esteem	31.23	5.23	17	40	−0.35	−0.54

According to Kenny (2008), who revises the steps for mediation analysis, first, it should be proven that the independent variable regresses over the dependent variable. If not, there is no relationship that may be mediated by a third one. In other words, if religiosity has no effect of over social adaptation, then mediation and moderation effects by a third variable are discarded.

Table 2 shows correlations between religiosity, self-esteem, and social adaptation. Religiosity is neither significantly related to social adaptation nor self-esteem. Moreover, predictive effects of religiosity over social adaptation and over self-esteem were also non-significant. Regression analysis of religiosity over social adaptation resulted in *adj. R^2^* = 0.003 (*p* = 0.13), while religiosity over self-esteem also resulted in *adj. R^2^* = 0.003 (*p* = 0.13). When the same regression analysis was conducted taking the grouping variable into account, results were still far from the significance threshold, *adj. R^2^* = 0.005 (*p* = 0.14) for religiosity over self-esteem, and *adj. R^2^* = 0.076 (*p* = 0.00) over social adaptation.

**TABLE 2:** Correlations for study variables.

**Variable**	**1**	**2**
1. Religiosity	–	
2. Social adaptation	0.07	–
3. Self-esteem	0.07	0.45**

*p < 0.05; **p < 0.01; ***p < 0.001.

In sum, according to this analysis, religiosity has an effect neither on social adaptation nor on self-esteem regardless if people live in vulnerable contexts or not. Since descriptive and simple predictive analysis gave sufficient evidence to reject our main hypothesis of a differentiated effect of religiosity over social adaptation through self-esteem, no further analysis was necessary.

### Exploratory Analysis

Religiosity is seemed to be related neither to self-esteem nor to social adaptation; however, a further exploratory analysis could uncover further relationships or patterns in this particular sample.

**FIGURE 2:**
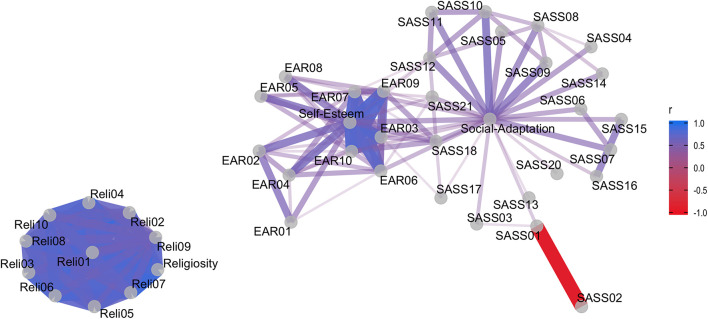
Correlation network among questionnaire items. Pearson’s *r* correlation between questionnaire items. SASS, Social Adaptation Self-evaluation Scale; EAR, Rosenberg’s Self-Esteem Scale; Reli, Santa Clara’s Religiosity Questionnaire.

One question that arose was if there were specific questionnaire items that could be related to indicators that measure distinct variables. The network plot (Figure 2) shows Pearson correlations among all items and total scores considered in our hypothesis. No items from the religiosity scale correlate to items or its score from other questionnaires, while social adaptation items and its score are related to some self-esteem items.

Group differences among variables were also analyzed. Both the control and the experimental groups have similar self-esteem [*M* = 31.2 (SD = 5.2) and 32 (5.7), respectively; *F* = 2.184 and *p* = 0.14] and religiosity levels [*M* = 21 (SD = 9) and 22 (SD = 9), respectively; *F* = 3.287 and *p* = 0.07, respectively], while they do differ in social adaptation scores, with higher scores in the control vs. the experimental group [*M* = 44 (SD = 6) and 41 (SD = 6), respectively; *F* = 38.38 and *p* = 0.00]. The latter is a foreseeable outcome if we consider that people who live in vulnerable contexts are characterized by having more socio economic struggles than the control group.

Despite these results, it is possible, however, that religiosity bolsters coping mechanisms that the concept and measure of social adaptation considered in this study fail to grasp. To further consider this idea, we conducted a regression analysis between religiosity and another variable, that according to the literature is related to social adaptation (Shahrier et al., 2016; Ortega et al., 2019) and that was measured in both the control and the experiment group: perceived stress.

Analysis showed that religiosity does not predict perceived stress either (*R*^2^ = 0.005, *p* = 0.06). If we consider group as an interaction term, there is also no moderation (β = 0.57, *p* = 0.73) or mediation (β = 0.06, *p* = 0.41) effects. Self-esteem, on the other hand, does predict perceived stress (*R^2^* = 0.324, *p* = 0.00) and social adaptation, respectively (*R^2^* = 0.205, *p* = 0.00).

Further mediation and moderation analysis strengthen the above results, that living in vulnerable contexts does not appear to determine the strength or direction of the relationship between self-esteem and social adaptation (moderation) (β = 4.255, *p* = 0.13), but it does seem to play a part in explaining the process through which both variables are related (mediation) (β = − 0.257, *p* = 0.00). In addition, living in vulnerable contexts seems to moderate and mediate the relationship between self-esteem and perceived stress (β = − 1.248, *p* = 0.00, β = 0.023, *p* = 0.00, respectively).

Finally, some studies that show a significant relationship between religiosity and variables related to coping and stress management use religiosity measures of religious practice and/or affiliation, which were also included in the survey for the control data collection. We analyzed if these variables, religious service attendance, and affiliation, are related to social adaptation or to self-esteem.

ANOVA analysis shows that there was no difference in self-esteem nor in social adaptation levels beteween different religious service attendance levels (*F* = 1.450 and *p* = 0.19, *F* = 1.289 and *p* = 0.26, respectively) or type of affiliation among people who do not live in vulnerable contexts (*F* = 0.806 and *p* = 0.52, *F* = 0.786 and *p* = 0.54, respectively). Thus, although this is an exploratory analysis and further analysis is needed to confirm non-relationship between these variables and self-esteem, these results give no reason to believe that religiosity has a generally positive impact on mental health variables as other studies have suggested.

## Discussion

This study wanted to test the idea of religiosity serving as a coping mechanism in people living under important amounts of stress, such as in the case of vulnerable populations. Specifically, we deduced from the literature that religiosity, through bolstering levels of self-esteem, would serve as a psychological tool so that people living in vulnerable contexts enlarge their chances for achieving social adaptation (in contrast to a control group of people from non-vulnerable contexts).

Surprisingly, and opposed to what several other studies suggest (Smith et al., 1964; Benson and Spika, 1973; Watson et al., 1985; Sherkat and Reed, 1992; Plante and Boccaccini, 1997; Greenway et al., 2003; Krause, 2003), religiosity was not associated with self-esteem. The results remained the same for both the experimental and the control group. Our study also shows that religiosity is not related to social adaptation directly or indirectly in either group, finding no evidence to believe that religiosity and social adaptation are related at all.

Regarding self-esteem, there were two mechanisms through which it was thought to be influenced by religiosity. First, it was proposed that religion provides people with social identity and meaning, through rituals and norms, generating a positive bias toward the ingroup, who would in consequence strengthen their self-esteem (Tajfel, 1981; Brown, 2000). Although our study suggests that religious strength does not affect levels of self-esteem, religiosity was measured using the Santa Clara’s Religiosity Scale, while future studies could use other measures of religiosity that may be better suited to test this specific mechanism such as religious affiliation or active participation in religious rituals.

The second mechanism suggests that self-esteem is an evolved cultural tool for dealing with the anxiety of facing the knowledge of death and uncertainty (Greenberg et al., 1997). This theory suggests that religiosity can offer a symbolic and/or literal immortality through a sense of the value of the person for a certain group or through beliefs in an afterlife. Although our study suggests that mechanism does not hold because self-esteem and religiosity show to be independent of one another, we did not evaluate if high levels of religiosity are related to lower levels of anxiety specifically, so further analysis would be needed to test this theory.

Since the acceptance of pre-registration of this study (2020), no new information has been found in the literature regarding the idea that religiosity is not related to self-esteem. On the contrary, recent studies continue to indicate that religiosity has an effect on self-esteem and that both variables are important for different aspects of well-being (e.g., Shafiee et al., 2020; Szcześniak and Timoszyk-Tomczak, 2020; Gábová et al., 2021; Sedikides and Gebauer, 2021). Thus, one may question why our main hypothesis did not hold even though there was a lot of research that suggested it would. One possibility is that most studies test their hypothesis on samples coming from western, educated, rich, and democratized countries (Henrich et al., 2010), while Chile does not entirely fit in this description. Thus, as such this study places boundaries on the presumed generalizability of the beneficial effects of religion for fostering self-esteem and social adaptation.

Another possibility is publication bias. As it is known, only recently efforts have been made to reduce the tendency of scientific journals to only publish studies where evidence sustains the stated hypothesis (Mehler et al., 2019; Allen and Mehler, 2019). Therefore, although there are several studies that suggest a relationship between religiosity and self-esteem and a relationship between religiosity and social adaptation, statistical effects found in them are low, and it is not possible to discard that they are part of the statistical error.

Our data do provide support for the idea that high levels of self-esteem foster well-being and related features across samples and groups with different characteristics (Dumont and Provost, 1999; Orth and Robins, 2014; von Soest et al., 2018) if we consider social adaptation as an element of well-being. These findings also support Neely-Prado et al.’s (2019) conclusions that self-esteem was the strongest predictor of social adaptation among several socio-affective variables in people who live in vulnerable contexts.

However, the results of this study make it clear that there is still much we do not understand about the interaction mechanisms that may operate between religiosity and different aspects of mental health and well-being, and that further research is still necessary. Specifically, regarding the current pandemic, some comments on the impact of religiosity, spirituality, and faith to compensate for stress associated with COVID-19 have started to appear (Dein et al., 2020; Koenig, 2020; Barmania and Reiss, 2021). Moreover, although some statistical findings have been published (Lucchetti et al., 2020; Pirutinsky et al., 2020), these are restricted to specific populations while cross-cultural and systemic studies could be more enlightening. At the same time, it is still unknown how this pandemic has disrupted self-esteem and social adaptation of people. Thus, further research on these mental health-related variables is highly encouraged as it can inform decisions of public support systems and health departments to better help people in tackling difficulties associated with isolation, high perception of infection risk, etc.

## Conclusion

In sum, our study has failed to provide support for the relationship between religiosity, self-esteem, and social adaptation, in a highly vulnerable and a non-vulnerable population in Chile. This finding casts doubt on the apparent cross-cultural universality of the idea that religion bolsters one’s self-esteem (Sedikides and Gebauer, 2021). It could very well be that at least a basic level of socio-economic security and stability is required at a national level before religion can yield any benefits to one’s well-being.

## Data Availability Statement

Raw data and analysis scripts are publicly available at the following repository: https://github.com/gorkang/jsPsychHelpeR-Neely.

## Ethics Statement

The studies involving human participants were reviewed and approved by the Research Ethics Committee of Universidad Adolfo Ibáñez. The patients/participants provided their written informed consent to participate in this study.

## Author Contributions

AN-P, GN, DH, and ME contributed to the conception and design of the study. AN-P and GN organized the database. AN-P and ME wrote the manuscript for pre-registration. AN-P wrote the final submission. GN designed the online survey platform and online data collection process. FH contributed to data collection and bibliographic revision. AN-P, GN, and DH ran the statistical analysis. DH takes primary responsibility for communication with the journal and editorial office during the submission process, peer review, and publication, is responsible for ensuring that the submission adheres to all journal requirements including, but not exclusive to, details of authorship, study ethics, ethics approval, clinical trial registration documents, and conflict of interest declaration, and is available post-publication to respond to any queries or critiques. All authors contributed to manuscript revision, read, and approved the submitted version, and theoretical approach of the study and its methodological design.

## Conflict of Interest

The authors declare that the research was conducted in the absence of any commercial or financial relationships that could be construed as a potential conflict of interest.

## Publisher’s Note

All claims expressed in this article are solely those of the authors and do not necessarily represent those of their affiliated organizations, or those of the publisher, the editors and the reviewers. Any product that may be evaluated in this article, or claim that may be made by its manufacturer, is not guaranteed or endorsed by the publisher.
